# A Giant Calcified Thoracic Hernia: A Case Report and Analysis of Complications

**DOI:** 10.7759/cureus.102210

**Published:** 2026-01-24

**Authors:** Iván M Ayala Collado, Antonio Vallejo-Estrella, Arturo De Jesús G Cano

**Affiliations:** 1 Neurological Surgery, Universidad de Guanajuato, León, MEX; 2 Neurological Surgery, Hospital General de León, León, MEX

**Keywords:** giant calcified hernia, lateral extracavitary approach (leca), myelopathy, postoperative complications, thoracic csf fistula, thoracic hernia, thoracic hernia complications

## Abstract

Giant calcified thoracic disc herniations are rare and represent a major surgical challenge due to their association with myelopathy, vascular compromise, and high perioperative morbidity. We report the case of a 62-year-old woman with progressive myelopathy secondary to a giant calcified thoracic disc herniation at the T7-T8 level. Surgical management via a lateral extracavitary approach was complicated by wrong-level discectomy, incomplete resection of the calcified disc, progressive spinal cord injury, cerebrospinal fluid fistula, infectious complications, and death. This case highlights critical technical pitfalls and emphasizes the importance of accurate level localization, vascular planning, and multidisciplinary management in complex thoracic spine surgery.

## Introduction

Thoracic disc herniations are defined as the displacement of intervertebral disc material into the spinal canal at the thoracic level and account for approximately 0.15% to 4% of all symptomatic disc herniations [1-3]. Compared with cervical and lumbar disc disease, thoracic disc herniations often present with subtle or slowly progressive symptoms, which may delay diagnosis and increase the risk of neurological compromise.

Lesions occupying more than 40% of the spinal canal are classified as giant thoracic disc herniations and are frequently associated with myelopathy, characterized by motor weakness, sensory disturbances, and gait impairment [2,4]. A significant proportion of these lesions are calcified, reflecting a chronic degenerative process with calcium deposition and adhesion to the dura mater. Calcified thoracic disc herniations constitute a particularly challenging subgroup due to their rigidity and the increased risk of incomplete decompression and dural injury [2,5,6].

Surgical intervention is indicated in patients with progressive neurological deficits or symptoms refractory to conservative management. Anterior and anterolateral approaches, including transthoracic and lateral extracavitary techniques, are generally preferred for giant or calcified thoracic disc herniations, as they allow ventral decompression while minimizing spinal cord manipulation [2-7]. Nevertheless, these procedures remain technically demanding and are associated with substantial morbidity.

Despite advances in surgical techniques, there is limited reporting on the cascade of technical and postoperative complications that may arise during the management of giant calcified thoracic disc herniations, particularly when initial errors such as wrong-level surgery occur. This case report aims to highlight critical technical pitfalls and clinical factors that can lead to catastrophic outcomes, providing practical lessons for spine surgeons and multidisciplinary teams.

This article was previously presented as a scientific poster at the XXIII annual congress of AMCICO (Mexican Association of Spine Surgeons) on September 20-23, 2023.

## Case presentation

A 62-year-old woman presented with a four-year history of progressively worsening thoracic back pain, associated with a gradual onset of lower extremity weakness over the preceding 12 months, which significantly worsened during the three months before admission. The pain was mechanical in nature, exacerbated by prolonged standing and ambulation, and rated as 7/10 on the Numeric Pain Rating Scale. She had been managed conservatively with nonsteroidal anti-inflammatory drugs, oral analgesics, and supervised physical therapy for approximately 18 months, without significant clinical improvement. Her medical history was notable for type 2 diabetes mellitus and systemic arterial hypertension, both diagnosed over 12 years earlier and controlled with pharmacological treatment. There was no history of trauma, fever, weight loss, or constitutional symptoms.

On neurological examination, upper extremity strength and sensation were normal. In the lower extremities, motor strength was graded as Medical Research Council (MRC) 4/5 bilaterally, predominantly affecting hip flexion and knee extension. Deep tendon reflexes were increased at both patellar tendons, and a sensory level was identified between the T10 and T12 dermatomes. The patient reported gait instability and required assistance for long distances, although she remained independently ambulatory. There was no bowel or bladder dysfunction.

**Figure 1:**
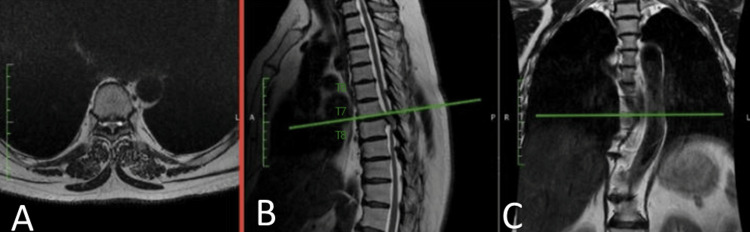
T2-weighted MRI showing a giant thoracic disc herniation at the T7-T8 level. Axial (A) view at T7-T8 demonstrating ventral spinal cord compression. Sagittal (B) and coronal (C) views illustrating the cranio-caudal extent of the lesion, with vertebral levels labeled for anatomical orientation.

**Figure 2:**
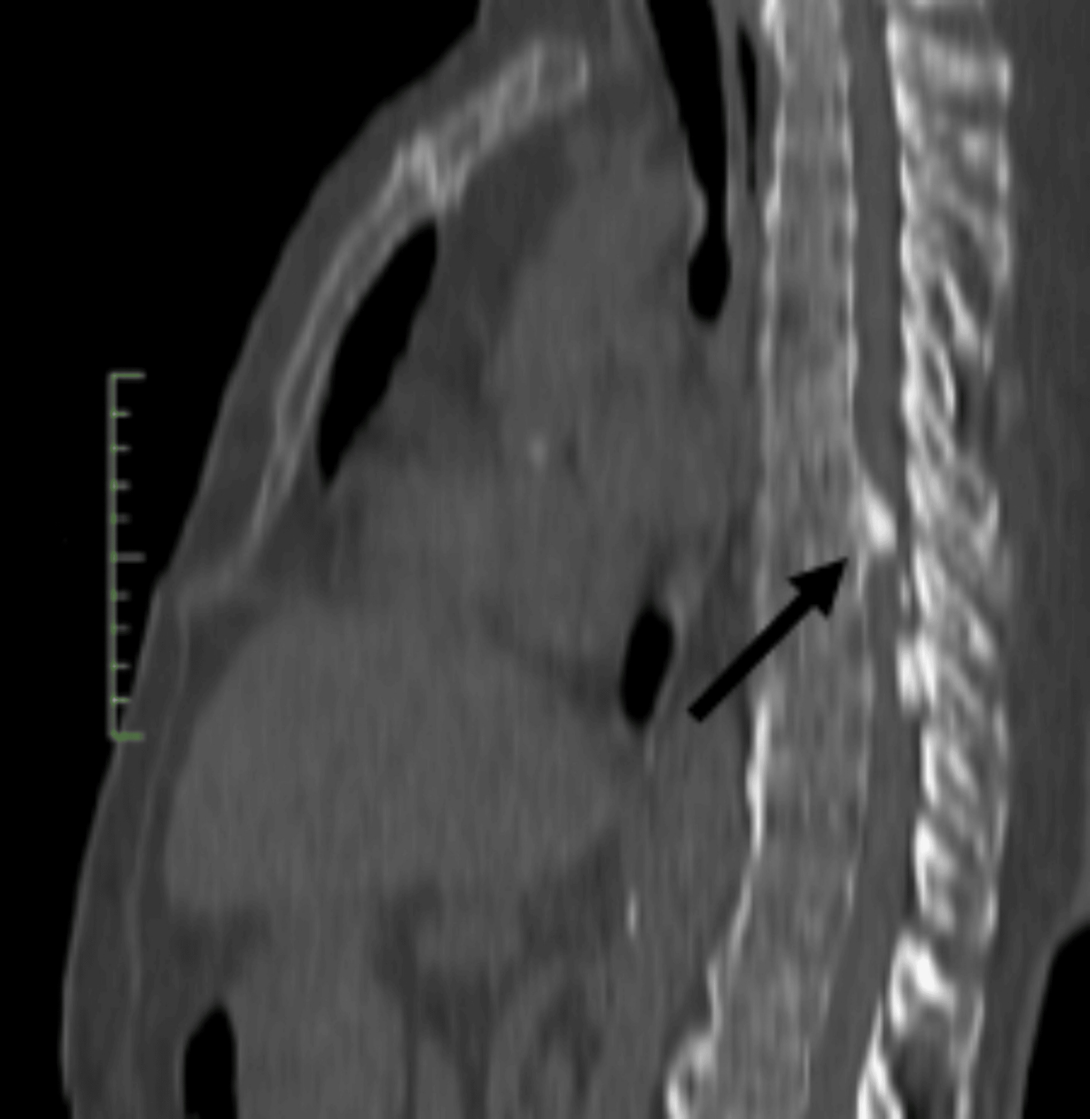
CT scan of the thoracic spine showing evidence of calcification of an extruded herniation at the T7-T8 level in a sagittal view (black arrow).

MRI and CT of the thoracic spine revealed a giant calcified disc herniation at the T7-T8 level, occupying more than 40% of the spinal canal, with significant ventral spinal cord compression and associated myelopathic changes (Figures 1, 2).

Given the progressive neurological deficit and failure of conservative management, surgical intervention was indicated. Preoperatively, a lateral extracavitary approach was selected to allow direct ventral decompression of the spinal cord while minimizing manipulation of neural elements. The main anticipated risks included dural injury, cerebrospinal fluid leakage, vascular compromise, and postoperative neurological deterioration, which were discussed with the patient before surgery.

After informed consent was obtained, a right lateral extracavitary approach was performed with the patient in the left lateral decubitus position. Partial resection of the seventh rib was required for exposure. A discectomy was performed, an interbody cage was placed, and anterior fixation was completed. An incidental pleural opening was identified intraoperatively, and a chest tube was inserted. Fluoroscopic level confirmation and intraoperative markers, such as sterile spinal needles, were not employed due to the lack of availability of the necessary equipment at our institution at the time of surgery.

Postoperatively, the patient reported improvement in pain (2/10). Neurological examination demonstrated no postoperative neurological deterioration, with preserved lower extremity strength (MRC 4/5 bilaterally), similar to the preoperative status, and no new sensory deficits.

However, postoperative CT revealed that the discectomy had been performed at the incorrect level (T6-T7), despite the use of fluoroscopic guidance. Unfortunately, no intraoperative images documenting this error were available for review.

**Figure 3:**
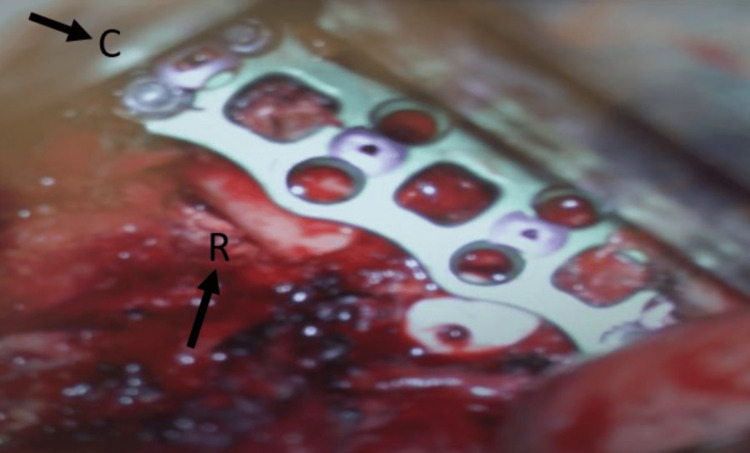
Intraoperative view showing anterior instrumentation with plate and screws and placement of an interbody cage at the T7-T8 level. The cranial (C)-caudal and left-right (R) orientation are indicated.

A second surgery was undertaken via a lateral extracavitary approach to address the correct T7-T8 level. The previously placed cage at T6-T7 was removed and replaced with an autologous bone graft. A T7-T8 discectomy was performed, an interbody cage was placed, and fixation was extended from T6 to T8 (Figure 3).

**Figure 4:**
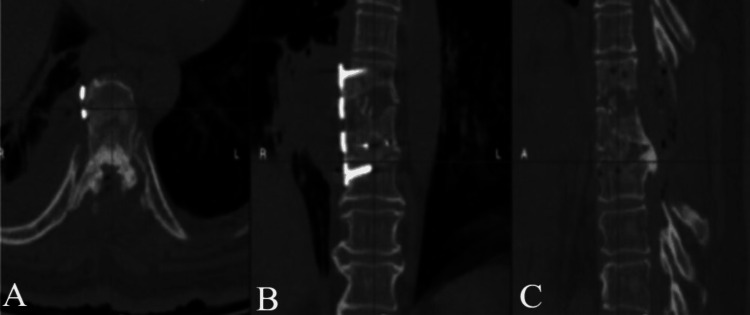
CT scan showing calcified extruded disc herniation at T7-T8, surgically treated with decompression via T7-T8 laminectomy in axial (A), coronal (B), and sagittal (C) plane.

Despite appropriate implant positioning, the patient developed progressive neurological deterioration, evolving to complete paraplegia. Urgent imaging demonstrated incomplete resection of the calcified disc fragment without evidence of a compressive epidural hematoma (Figure 4).

A third procedure was performed via a posterior thoracic approach with T7-T8 laminectomy. No neurological improvement was observed. Several days later, the patient was readmitted with respiratory compromise, and imaging studies revealed a thoracic cerebrospinal fluid fistula.

**Figure 5:**
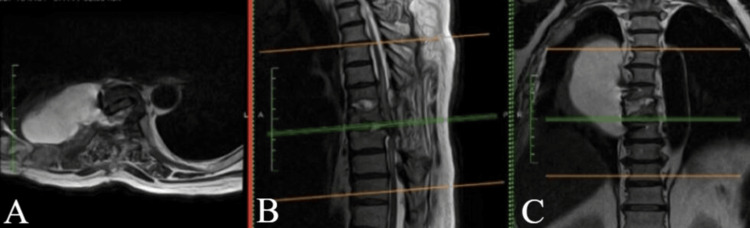
T2-weighted MRI, identifying cerebrospinal fluid fistula likely due to a T7-T8 defect in the axial (A), sagittal (B), and coronal (C) plane.

Neurological deficits remained unchanged during four days of postoperative observation. The patient was discharged due to the established clinical status, with an outpatient follow-up appointment scheduled in two weeks. She was readmitted five days after discharge due to respiratory deterioration, and a cerebrospinal fluid fistula was confirmed on MRI (Figure 5).

A fourth surgery was performed in conjunction with the thoracic surgery team through a thoracotomy approach. Two small pulmonary lacerations were repaired, a dural substitute was placed, and a subarachnoid drain was inserted. Cerebrospinal fluid cultures grew *Candida albicans*, and antifungal therapy with amphotericin B was initiated. Despite aggressive multidisciplinary management, the patient progressed to septic shock and died.

## Discussion

Giant calcified thoracic disc herniations represent one of the most technically demanding entities in spine surgery. Disc calcification and dural adhesion significantly increase the risk of incomplete decompression, dural tears, and neurological deterioration [2,6]. In the present case, the rigid and adherent nature of the calcified disc limited complete decompression during the second procedure, contributing to persistent spinal cord compression and irreversible neurological injury.

Wrong-level surgery represented a critical turning point in the patient’s clinical course. Although fluoroscopic guidance was used, standard risk-reduction strategies such as intraoperative markers or sterile spinal needles were not employed due to the lack of available resources at our institution. This limitation underscores the importance of adapting surgical safety protocols to local resource constraints and reinforces that, even in limited-resource settings, alternative strategies for accurate level confirmation must be systematically implemented to prevent catastrophic errors [6,8-11].

Although spinal cord ischemia was neither suspected nor confirmed in this case based on clinical or imaging findings, it remains a well-recognized and devastating complication associated with anterior and anterolateral thoracic approaches. Injury to the artery of Adamkiewicz, which most commonly arises between T8 and L2 and originates from the left side in approximately 80% of cases, may result in irreversible neurological injury [12]. Its inclusion is relevant in the context of giant thoracic disc surgery, particularly given the proximity to critical segmental vessels, and highlights the importance of preoperative vascular imaging and hemodynamic optimization [12,13].

Cerebrospinal fluid leakage is more frequently observed in calcified thoracic disc herniations due to dural thinning and firm adhesion to the calcified disc shell. In the thoracic region, cerebrospinal fluid leakage may evolve into a subarachnoid-pleural fistula secondary to negative intrathoracic pressure, often requiring surgical reintervention [2,6]. In this case, the cerebrospinal fluid fistula and subsequent fungal infection represented terminal complications that ultimately led to septic shock and death.

This case underscores the importance of meticulous surgical planning, careful intraoperative technique, institutional resource awareness, and thorough preoperative counseling regarding the potential for catastrophic outcomes in patients with giant calcified thoracic disc herniations.

## Conclusions

Giant calcified thoracic disc herniations are associated with a high risk of severe complications despite appropriate surgical management. In this case, wrong-level surgery due to limited intraoperative localization resources, incomplete decompression of a rigid calcified disc, and subsequent cerebrospinal fluid fistula with infectious complications proved to be decisive factors in the fatal outcome. Accurate level localization adapted to available resources, meticulous decompression techniques, and early recognition and management of postoperative complications are essential to reduce morbidity and mortality in these complex cases. This case underscores that technical precision and system-level preparedness are equally critical in the management of giant calcified thoracic disc herniations.
